# Editorial: Introducing *NAR Cancer*

**DOI:** 10.1093/narcan/zcz001

**Published:** 2019-12-16

**Authors:** William S Dynan

**Affiliations:** Departments of Radiation Oncology and Biochemistry, Winship Cancer Institute, Emory University, Atlanta, GA 30324, USA

*NAR Cancer* is a new Open Access journal that publishes research and review articles at the intersection of the nucleic acids and cancer fields. We are a daughter journal to the highly successful *Nucleic Acids Research* (*NAR*), which is now in its 47th year of publication and ranks consistently among the top 5% of its peers in the biochemistry and molecular biology category. We hope to carry forward the best traditions of *NAR*, including management by and for working scientists, while providing a venue to highlight cancer-specific studies. *NAR* receives more than 4000 submissions each year and publishes more than 1000. A move to spin off a more specialized journal was timely and necessary. Whereas *NAR* is devoted to the whole broad field of nucleic acids and the molecules with which they interact, *NAR Cancer* will focus on high-quality studies with special significance to the cancer field. We encourage direct submissions, but we also have the facility for seamless transfer of articles originally submitted to *NAR*.

In addition to scientific rigor and excellence, our criteria for acceptance will include potential impact in understanding the causes of and providing cures for cancer. Although our emphasis is mechanistic, studies that feature preclinical validation or clinical correlates are welcome. We are open to articles that focus on a particular type of cancer or that focus on a translationally relevant subject. The article categories in *NAR Cancer* mirror those in *NAR* and span all aspects of cancer-related nucleic acids research, including cancer computational biology and data resources, DNA damage sensing and repair, gene regulation and chromatin, genomics, methods, nucleic acid-based therapeutics, and structural biology.

This is a remarkable time in the cancer field. In the span of one lifetime, we have progressed from a rudimentary to a rich and sophisticated understanding of carcinogenesis and cancer genetics. In his 1966 Nobel Lecture, Peyton Rous spoke of the ‘proliferative, rampant, predatory, and ungovernable’ nature of tumors. He went on to say, ‘despite more than 70 years of experimental study they remain the least understood. This is the more remarkable because they can be evoked at will for scrutiny by any one of a myriad chemical and physical means which are left behind as the tumors grow’ (1). The picture could not be more different today. We now understand the centrality of nucleic acids in all aspects of carcinogenesis and cancer biology. Defects in DNA damage signaling and repair, resulting in mutations, are central to cancer etiology. Altered chromatin structure, epigenetic marks and transcriptional regulation are key requirements to sustain the cancer phenotype. Genome-wide analyses and targeted therapeutics afford ever-more-precise approaches to therapy. Our hope is to capture the best of this new era in the pages of our journal.

Before assuming my current role as Editor-in-Chief of *NAR Cancer*, I was an Editorial Board member and Executive Editor at *NAR* for more than 25 years, and thus, more than half the time of the journal's existence. At *NAR*, we devoted a great deal of time and discussion to emerging trends in science and publishing. We were one of the first major print biology journals to embrace a fully Open Access publishing model, on the premise that the same public that pays for scientific research should be able to freely access the fruits of that research. Under an Open Access model, readers at small institutions, start-up biotechnology companies and in the developing world enjoy the same access as those at major research centers. We will continue that fully Open Access tradition at *NAR Cancer*. We also embrace the concept of Open Science. Like *NAR*, *NAR Cancer* will require deposition of large-scale data sets in appropriate repositories concurrent with the review process and, indeed, we encourage the deposition of all other supporting data, including unprocessed images and underlying numerical data in support of display items. Although challenging, we hope to carry on the tradition of rapid peer review (which at *NAR* averages about 24 days from submission to first decision), and of rapid publication of a fully copy-edited article soon after acceptance. Additionally, as working scientists, the editors understand the time and resources involved in performing reviewer-suggested experiments, and we are committed to maintaining a dialog with our authors in this respect.

One of my first tasks upon assuming the Editor-in-Chief position was to form an Editorial Board. Nearly everyone we approached agreed to serve. At present, our Editorial Board is composed of 45 distinguished scientists, drawn from four continents, and representing a variety of disciplines, stages of career and perspectives. Over the coming months, we hope to increase the depth and diversity even further.

I would like to acknowledge our Managing Editor, Martine Bernardes-Silva (who performs a similar role at *NAR*), our four Associate Editors, Yamini Dalal (US National Cancer Institute), Subho De (Rutgers, the State University of New Jersey), Steven A. Johnsen (Mayo Clinic) and Jean-Yves Masson (CHU de Québec-Université Laval), and all the others who have agreed to help make the journal a success. The most important factor in this success will, of course, be support from the broader cancer community. We hope that you will take a chance on us: that you will contribute to the journal, assist with peer review, and read and cite our content. Please help demonstrate that there is a place for a new peer-edited, rapid review, non-profit, Open Access journal: *NAR Cancer*.
